# SYSTEMS AGING AND REJUVENATION

**DOI:** 10.1093/geroni/igac059.075

**Published:** 2022-12-20

**Authors:** Vadim Gladyshev

**Affiliations:** Brigham and Women's Hospital, Boston, Massachusetts, United States

## Abstract

What is aging? When does it begin? How to control lifespan? Can we rejuvenate organisms in addition to slowing down the aging process? There is no consensus on these questions, but recent developments in the field may allow to address these questions. In particular, DNA methylation of defined sets of CpG dinucleotides emerged as a critical and precise biomarker of the aging process. Multi-variate machine learning models, known as epigenetic clocks, exploit quantitative changes in the methylome to predict the age of bulk tissue with remarkable accuracy. Additionally, the first epigenetic aging clock that works at the level of single cells has been developed. Together with advances in genomics, transcriptomic longevity signatures and intervention strategies, these tools support quantification and manipulation of the aging process. Moreover, these tools may also be used to assess the possibility of age reversal. Several types of rejuvenation have been described, including the recently discovered process of early embryonic rejuvenation, culminating in ground zero, marking the beginning of organismal aging.

